# Is past life regression therapy ethical?

**Published:** 2017-12-02

**Authors:** Gabriel Andrade

**Affiliations:** *Department of Ethics and Behavioral Science, Xavier University School of Medicine, Oranjestad, Aruba.*

**Keywords:** *Past life regressions*, *Psychiatry*, *Reincarnation*, *Medical ethics*

## Abstract

Past life regression therapy is used by some physicians in cases with some mental diseases. Anxiety disorders, mood disorders, and gender dysphoria have all been treated using life regression therapy by some doctors on the assumption that they reflect problems in past lives. Although it is not supported by psychiatric associations, few medical associations have actually condemned it as unethical. In this article, I argue that past life regression therapy is unethical for two basic reasons. First, it is not evidence-based. Past life regression is based on the reincarnation hypothesis, but this hypothesis is not supported by evidence, and in fact, it faces some insurmountable conceptual problems. If patients are not fully informed about these problems, they cannot provide an informed consent, and hence, the principle of autonomy is violated. Second, past life regression therapy has the great risk of implanting false memories in patients, and thus, causing significant harm. This is a violation of the principle of non-malfeasance, which is surely the most important principle in medical ethics.

## Introduction

Past life regression is a technique that attempts to use hypnosis in order to recover memories from previous lives. According to past life regression therapists, many mental health issues that patients experience may have their origins in traumatic experiences of past lives. Thus, through hypnosis, practitioners take the patients back in time (regression) (1). This regression could be to periods of their infancy, but also to periods of their gestational development, or periods before they were born with their current body, but their soul may have been embodied in another body, i.e., past lives.

Past life regression therapy, therefore, assumes the reality of reincarnation. Based on this assumption, practitioners believe that various mental disorders can be treated by addressing the events that an individual went through before he or she was born in this life.

Mainstream psychiatry has traditionally rejected this assumption. However, some high profile practitioners in the field have upheld past life regression therapy, and actually incorporate it in their medical practice as a therapeutic technique. Perhaps the most prominent promoter of this therapeutic approach is psychiatrist Dr. Brian Weiss. In a series of bestselling books, Dr. Weiss has recommended hypnosis for patients, in order to overcome phobias. These phobias, Dr. Weiss believes, go back to experiences from previous lives (2). By going back to those experiences through hypnosis, the patient confronts his/her fears, and ultimately, becomes desensitized to his/her original fears. Dr. Weiss’s therapeutic approach has gained notoriety, because at first, he did not believe in reincarnation. According to his testimonies, as he encountered patients that allegedly gave precise details of their past lives, Dr. Weiss came to change his mind. In the public’s view, Dr. Weiss’s initial skepticism renders him some professional credibility; he came to believe in reincarnation and the efficacy of past life regressions not due to some whacky previous religious beliefs, but rather because evidence from his medical practice led him to it. 

Although the medical establishment does not favor these procedures, there is a high demand for them in the general population. According to some estimates, 25% of the American population believe in reincarnation, and that figure is surely higher in countries with religions (Buddhism, Hinduism) that give karma and reincarnation a strong relevance in their belief systems (3). 

For the most part, professional psychiatric associations have refused to offer support to these techniques, but they have not gone further in calling into question the ethics of such procedures. In this article, I shall evaluate the ethics of past life regression, by considering three of the most important principles in medical ethics; autonomy, beneficence, and non-malfeasance. I will address three points: 1) is reincarnation even conceptually possible? 2) Does the evidence really support the reincarnation hypothesis?; and 3) Is past life regression therapy harmless?

***Is reincarnation even conceptually possible?***

Reincarnation beliefs are quite ancient. In Ancient Greece, Pre-Socratic philosophers speculated about the soul travelling from one body upon death to another newly born. These beliefs were not particularly important in Western societies, but in the East, they did become quite prominent, first, during the Vedic Period, and then, with some of the great religious reforms by Buddha and Mahavira.

Yet, even from very early times, some philosophers realized that, regardless of the actual evidence, there are some conceptual objections to reincarnation. First, there is the problem of population growth. The human population has increased continuously throughout history. In 8,000 BCE, the human population was at about five million; today, it is at about six billion. Now, if the doctrine of reincarnation is true, the number of souls is constant. For, when someone dies, her soul does not become extinct, and when someone is born, her soul is not new, as it is travelling from another body. However, if the number of souls is constant, how can we explain the increase in the number of bodies? Presumably, in the year 8,000 BCE, there were five million soles in the world. Today, presumably, there are six billion souls. Where did these additional souls come from? 

In truth, this objection is not formidable. According to the reincarnation doctrine, there is no reason to assume that no new souls can be created. Even if the number of souls is constant, some of them could have a disembodied existence while they wait for new bodies to come into existence, as the population increases. Furthermore, if, as Hinduism teaches, reincarnation need not only take place amongst human beings, then, the remaining souls could also be embodied in animals, while they wait for new human bodies to be born.

However, there are some other important conceptual objections. If reincarnation is real, it is nevertheless true that most people do not remember their previous lives. Now, without memories from previous lives, how can we allege that someone is the same person (or, as philosophers would phrase it, *numerically identical*) as the one who lived in a previous life? For the sake of argument, we may admit that the criterion of personal identity (i.e., how do we make sure that a person at a given time is the same person at another time?) is not the body (although, some philosophers do insist that the only possible criterion is the body, for reasons that I shall not delve into). Under this assumption, one need not have the same bodily continuity in order to be considered the same person, as long as there is at least some psychological continuity. The philosopher John Locke famously argued that if a prince would one day wake up in the body of a cobbler, but would keep his memories as a prince, then, he would still be the prince (4).

Nevertheless, the problem with reincarnation is that there is precisely no psychological continuity, as most people do not remember their previous lives. By the psychological criterion of identity, then, we cannot be someone of whom we have no memories. Defenders of reincarnation claim that we have no memories from our earliest infancy, but that does not mean we are not the same person. Furthermore, patients with memory impairment (such as patients with Alzheimer’s disease) have few, if any, memories of their life, but again, that does not mean they are not the same person.

However, philosophers have usually pointed out an important caveat regarding the psychological identity criterion: if there is a chain of memories from one stage to a later stage, then, identity is preserved. Thus, for example, an elderly general may have memories of his time as a young officer, and this officer had memories of his childhood, then, we can say that the elderly general is the same person as the child. Yet, in the case of reincarnation, there is no such chain.

Apart from these conceptual problems specific to reincarnation, there are also conceptual problems regarding the existence of souls. The doctrine of reincarnation assumes that souls exist, as a person’s soul abandons his/her body upon death, and enters another body at the moment of birth. However, if the soul is a mysterious nonmaterial substance, how does it interact with matter? Back in the 17^th^ Century, philosopher Rene Descartes was aware of this problem, and he argued that soul and body interact in the pineal gland (5). Now we know that this is wrong; the pineal gland has no cognitive function. But even if it did, the problem remains: how does a nonphysical substance make its entry into the physical universe and become a causal agent?

***Is there evidence for reincarnation?***

As early as the times of Plato, some philosophers have tried to offer evidence that seems to support the reincarnation hypothesis. According to a famous argument laid out in Plato’s *Phaedo*, we have knowledge that we could not possibly acquire in this lifetime. Plato was intrigued by the abilities of some people to do certain things that they never learned to do. In Plato’s estimation, learning is actually a form of reminiscence: in education, while we are stimulated by our teachers’ questions, we come to remember things from our previous lives (6).

It is no doubt true that we have innate knowledge and innate mental contents. Nevertheless, as opposed to what Plato believed, that does not imply that it comes from previous lives. Actually, our brains may be genetically hardwired for certain mental traits, and some specific innate knowledge. One need not have been bitten by a snake in a previous life in order to innately fear snakes. The fear of snakes was very likely advantageous in the African Savannah, and thus, that fear is probably encoded in our genes. 

Defenders of reincarnation also point out déjà vu experiences. They are the strange feeling some people get when they encounter a situation for the first time, but they have the sensation that they have already lived through it. But again, we must not rush and jump to conclusions. It has been proposed that déjà vu experiences are due to a mismatch in the timing of sensorial information processing; it is possible that one of the brain’s hemispheres assimilates information, and a short time later, the other hemisphere assimilates the information. In such a scenario, the person would believe that he/she is reliving incident that, in fact, only occurred a few milliseconds ago. 

Déjà vu experiences may also be explained as instances of cryptomnesia, i.e., when a person stores some sensorial datum in their memory, but it soon disappears from conscious memory. These memories may remain hidden in the person’s mind, and they may again be activated in a similar situation, without the person having a clear understanding of those memories.

Probably the strongest body of evidence in favor of reincarnation comes from the collection of cases by a prominent American psychiatrist, Dr. Ian Stevenson (7). Stevenson was skeptical about past life regressions, but he did believe that the etiology of many mental disorders can be traced back to unsolved conflicts in previous lives. In particular, Dr. Stevenson was interested in gender identity disorders (the DSM 5 now calls this disorder “Gender Dysphoria”); in his estimation, many of these cases are due to the fact that, in a previous lifetime, the patient belonged to another gender group.

Stevenson’s main area of research was the phenomenon of children who allege to remember previous lives. He collected a considerable amount of cases in India, Sri Lanka, Turkey, Brazil, Lebanon, and the US. In most of those cases, children would not behave as expected. Many of these children would speak rudely to their parents, for they believed themselves to be their own grandparents. 

According to Stevenson’s reports, other children developed attitudes and interests not in accordance with their age; Stevenson interpreted this as coming from a previous life. Some children developed particular phobias, with no particular experience to activate them. Again, he speculated that these phobias came from traumatic events in previous lives (8). 

In the majority of cases investigated by Stevenson, children alleged to be the reincarnation of some member of the same family. In other cases, however, children alleged to be the reincarnation of persons with the family of whom the child’s family was not acquainted. Some of these children were capable of giving details regarding their previous lives, without any possibility of the child having known these details through other means. In one case, a boy from a village claimed to be the reincarnation of a man who died some decades ago, and lived in a distant village. The child had never abandoned his own village, and therefore, never had the opportunity to know the details about the man’s life.

Stevenson also paid attention to birthmarks and birth defects in children who remembered past lives (9). In most of these cases, children claimed to be people who died violently, and their birthmarks would correspond to the wounds they had as a result of the injury that caused their death. Stevenson claimed to have found a considerable number of this type of cases.

Stevenson’s studies are quite extensive, and in this brief space, I cannot discuss the details of each case. However, I can point to some methodological flaws that cast a big shadow over his findings.

Even if he claimed not to have definitive data supporting the reincarnation hypothesis, Stevenson seemed to have a preconceived idea, and he just sought the way to confirm it. He may have been guilty of confirmation bias. All Stevenson ever did was to look for cases that seemed to confirm his initial preconceived hypothesis, and ignore the massive amount of cases that do not fit his initial expectation. The number of children who did *not *remember past lives far exceed the number of children who did, but Stevenson never took this into account. Whenever a case did not fit his preconceived idea, he just moved on to the next one that, apparently, did fit into his mold.

The main problem with Stevenson’s research, then, is that his hypotheses are not falsifiable. According to philosopher Karl Popper, this is the definite criterion separating science from pseudoscience (10). No possible counterexample can ever refute pseudoscience, because pseudoscience always has a way of accommodating via *ad hoc *hypothesis. This was the case with Stevenson’s research. Every time he was presented with evidence that seemed to refute his hypothesis, he would just move to another case.

Stevenson was apparently apt at verifying his hypothesis (hence his massive data), but not at confronting evidence contrary to his theories. In his research, unlike true scientific research, there was no possible counterexample that he would be willing to take as a refutation of his claims. The question “what evidence would be enough for you to change your views?” was left unanswered by Stevenson.

Another important criterion in the philosophy of science is predictability. As opposed to pseudoscientific theories, scientific theories can make predictions. Science assumes regularity in nature. Thus, if science pretends to know the laws of nature, it should have the capacity to elaborate predictions on the basis of its knowledge of nature. 

Stevenson’s theories, however, have no predictive value. For example, if, as Stevenson claims, a violent death will lead to a reincarnation in which the child will have birthmarks, then, we should at least expect some predictions about particular birthmarks in future incarnations. Yet, Stevenson did not provide any clue on future specific birthmarks.

It is true that Stevenson never claimed that his data is definitive, and it is also true that science requires an open mind to consider possible cases. Apart from Stevenson’s studies, little research has been done on the possibility of reincarnation, and the jury may still be out. However, Stevenson’s data is too weak to even suggest reincarnation, and his collection methods are very questionable.

Stevenson did not speak the languages of the societies in which he carried out his studies. He relied on local interpreters, and this allowed for various cases of corruption. In many of the countries where Stevenson did his research, there is considerable cultural expectation when it comes to cases of reincarnation. The interpreters, consciously or not, could have offered data confirming reincarnation, even though it may not have been the subjects’ original testimonies. Stevenson should have been careful to independently validate his interpreters’ translations, but he never did that. His research does not present audio recordings or even transcriptions of interviews in the informants’ original languages. In fact, Stevenson had some interpreters who were later found to be fraudulent (11); Stevenson himself admitted his interpreter’s dishonesty in some aspects, but he still trusted his translations. That is extremely naïve and scientifically unacceptable.

There are other graver problems. In the vast majority of Stevenson’s cases, children claimed to remember the lives of people who were either a part of the child’s family or close to them. In addition, Stevenson’s questions induced the informants to give the information that he wanted in the first place (12). Furthermore, the interviewing time was extremely short, which again, seems to suggest that Stevenson was more interested in getting the information that fit his preconceived ideas, and after he got that, he would not investigate further.

Stevenson also had the habit of not only incorporating the children’s testimonies, but also the adults’ interpretations into his reports. In most cases, adults would favor the reincarnation hypothesis, so their biases were incorporated into the data. In fact, Stevenson rarely spoke to the children, in part because the children were too shy to talk to a Western researcher. Adults spoke for the children, and again, this allowed for the adults’ biases to come through. Some parents even knew the relatives of the person whose life the child allegedly remembered; hence, the probability of the child getting information from them was increased. As a matter of fact, only in a small proportion of cases, the child’s family did not know the deceased person’s family.

Generally, Stevenson received the news that in some village, a child claimed to remember past lives, and then, he went to investigate the case. Between the time that he received the news and he finally reached the village, a lengthy time (three weeks to two years) passed. During that time, the child’s family could have met the deceased person’s family, and they could have gathered information that ultimately reached the child. By the time Stevenson arrived, the child would be able to give some specific details, and of course, they would not come from the child’s alleged memories, but rather, from the information that came as a result of the families’ encounters.

Furthermore, the fact that the majority of cases investigated by Stevenson were violent deaths also raises some suspicions. Violent deaths are much more publicized than nonviolent ones. That increases the availability of information, and hence, the probability of the child gaining details on the deceased person’s life.

The fact that most of these cases take place in countries where reincarnation is a mainstream religious belief also raises suspicions. The child’s family may already be conditioned to believe that the child does remember a previous life, and they may actually encourage such beliefs in the child. Any small gesture coming from a child may be interpreted as a sign of remembering past lives, and this further serves as feedback for the child to elaborate on his claims in fulfilment of the parents’ expectations.

In the cases collected by Stevenson, there also seemed to be a correlation between the culture’s beliefs and the way the cases develop. For example, in cultures where it is not accepted that someone may reincarnate in the opposite sex, no child would remember a previous life in the opposite sex. In matrilineal cultures, children mostly remembered the lives of matrilineal relatives, whereas in patrilineal cultures, children remembered the lives of patrilineal relatives (13). There is also the issue that many of Stevenson’s cases were from India. This raises the suspicion that some children may claim to remember the lives of people from upper castes as a way to scale upwards in the caste system. 

All these methodological problems come up as a result of a central flaw in Stevenson’s research: all he really did was to collect anecdotes. Anecdotal evidence may be useful at first, but it is not enough to build a strong case for a hypothesis. Furthermore, as Carl Sagan famously claimed, extraordinary claims require extraordinary evidence. Reincarnation is an extraordinary claim, but Stevenson’s investigations are not extraordinary evidence. In Stevenson’s research, there are no controlled experiments.

As for the extraordinary talents developed by children, reincarnation is not the only possible explanation. Talents (artistic, academic, and etc.) have various heritability rates, but basically most of them do have a genetic basis. Some defenders of reincarnation claim that some children with extraordinary talents come from families without those talents. However, that is not a good enough argument, for it ignores a basic law in Mendelian genetics: a given trait may disappear in one generation and reappear in another. The parents may carry the dominant unexpressed variety of a gene for a specific talent. 

It has been frequently claimed that Mozart must have been the reincarnation of a great musician, for, how can someone at such a young age develop those musical skills? Again, there is no need to appeal to reincarnation: it is quite possible that Mozart may have had an acute auditory cortex, which allowed him to develop his impressive musical talents at an early age (14).

Stevenson always claimed that the most important cases were the ones in which children had birthmarks supposedly coinciding with the wounds that came as a result of the deceased person’s violent death. But again, all this evidence is just anecdotal. The deceased person’s body had already decomposed, so there did not seem to be a good opportunity to analyze the details of the wounds and compare them to the birthmark. Stevenson only relied on testimonies and photographs; both types of evidence are highly susceptible to fraud.

Moreover, once again, in these cases, Stevenson arrived too late. This delay allowed for the possibility of the child’s family, by contemplating the child’s birthmark, investigating who in the village may have died with similar wounds. The families may have established contact, and the child may have been provided with information about the deceased person. By the time Stevenson arrived, the child may have elaborated his/her alleged memories feeding on that information.

Furthermore, if reincarnation is just about the transmigration of souls, how exactly do marks appear on the body? Neither Stevenson nor any other defender of the reincarnation hypothesis has ever given a satisfactory response to this important question. Once again, we face the problem of interaction between material and nonmaterial substances. 

***Is past life regression therapy harmless?***

Despite the fact that, as I have argued, the reincarnation hypothesis faces some tough conceptual problems and the empirical evidence in its favor is very weak, some psychiatrists do insist on using past life regression therapy to treat some mental disorders, specially specific phobias (15). 

The movement to use past life regression as therapy in psychiatry began with a famous case, that of Bridey Murphy, in the 1950s (16). In 1952, an amateur hypnotist, Morey Bernstein, hypnotized Virginia Tighe, a woman from the State of Colorado in the US. Under hypnosis, Tighe assumed the personality of one Bridey Murphy, allegedly an Irish woman from the 19^th^ Century. Tighe had never travelled to Ireland, but in the hypnosis sessions, she spoke with a British accent, and surprisingly, she gave precise details about Irish life in the 19^th^ Century. 

Bernstein wrote a book about this case (17), and the details provided in the book made some scientists think that, indeed, Tighe was the reincarnation of Murphy. However, as the case drew the attention of the media, some reporters went to Ireland to investigate, and found out that Tighe´s details had some flaws. The registry details of the town where Murphy had allegedly been born did not match the local records. Tighe claimed that in her previous life she lived in a wooden house, but in fact, in her alleged hometown there were no wooden houses.

It turned out that one Bridie Murphy Corwell had been a neighbor to Tighe during her childhood. It was highly probable that she heard stories about Ireland from this neighbor, and this information came out during her hypnosis sessions, although she believed them to be part of a past life.

The case of Bridey Murphy is very illustrative of what really goes on during past life regressions. During these procedures, cryptomnesia is very significant. Cryptomnesia, let us recall, is a process during which a subject records some information in his/her mind, but this information remains “hidden” in the unconscious. Under special circumstances (such as hypnosis), the subject may think that he/she is once again living a particular experience, when in fact, it is just a hidden memory. For the most part, memory is a selective process; we daily apprehend an enormous amount of sensory data, and we must discriminate on the basis of relevance. We normally choose what we desire to remember. Some data is then deleted, but some unwanted data nevertheless is retained. When this information reappears, we believe it to be a new experience, or in cases such as Bridey Murphy´s, we believe it to be reminiscence of past lives.

In the case of hypnosis and past life regression, the fact that the hypnotized subject is in a state of suggestibility increases the probability of the occurrence of cryptomnesia. In hypnosis, the hypnotist easily manipulates the subject´s mental state. Thus, in hypnosis, the hypnotist may easily induce the subject to assume the role of some character in a previous life. The hypnotist may even do this unconsciously, by asking leading questions that, under a state of suggestibility, the hypnotized subject follows and complies with the initial request. For example, the hypnotist may state: “Go back to your life as a soldier in the American Revolution”, and taking cue from the hypnotist, the subject may begin to behave in a manner that she believes appropriate for soldiers during the American Revolution on the basis of some memories stored in history class during her schooldays. The hypnotist may be excited by this response, he may ask even more leading questions, and ultimately, the hypnotized subject may provide some apparently vivid details of some battle, again on the basis of some previous educational experience (reading a book, watching a movie, and etc.). 

Past life regression, as with most psychotherapies, can provide some good results. Talking about one´s problems will offer some form of relief. Thus, even if reincarnation is not real and past life regression is just a role-playing game in which the patient follows the suggestions of the hypnotist, is it not ethically acceptable? If the bottom line is helping patients, why should there be any moral objections to this procedure?

Let us consider the three important principles of medical ethics (beneficence, autonomy, and non-malfeasance) in order to answer this question. It is undoubtedly true that past life regressions are performed with the intention of helping patients, and indeed, many patients do find some relief undergoing these therapies. In such a manner, the principle of beneficence (the promotion of the wellbeing of others) is honored.

Past life regressions are not forced on patients. They are always done with the consent of the patients. Therefore, apparently, the principle of autonomy is also honored. Yet, there is concern as to how informed patients are when it comes to these procedures. In order for the principle of autonomy to be truly honored, there must be informed consent. When doctors do not explain to patients that there is no scientific evidence for reincarnation (and that reincarnation even faces some difficult conceptual problems), patients are not fully informed. With such a lack of information, there can be no true consent, and hence, the principle of autonomy is compromised.

Most ethicists agree that the most important principle of medical ethics is actually non-malfeasance (First, do no harm) (18). Even if a patient asks for a procedure, if that procedure is likely to cause harm, the physician should abstain from performing it. Past life regressions can have good outcomes, but they also carry some significant risks. Those risks far outweigh the possible beneficial consequences of this type of therapy.

The greatest risk in past life regressions is that the hypnotist may implant false memories in the subject, and due to suggestibility, the subject may come to feel them as quite real. This implantation of false memories need not even be intentional. The therapist may ask a question, such as “Were you ever a soldier in the American Revolution?”, and the subject, inasmuch as she desires to fulfill the role that the therapist apparently expects of her, elaborates on the details. If the hypnotist asks a leading question that may suggest a traumatic experience to the subject, even if that traumatic experience never actually happened, it may actually become quite real for the hypnotized person.

In fact, this danger became especially acute during the Satanic Ritual Abuse moral panic of the 1980s in the US. Hundreds of patients underwent hypnosis in order to recover memories of alleged sexual and ritual abuses during their infancies. A thorough FBI investigation was carried out, and no evidence whatsoever was found to support the allegations of sexual and ritual abuse (19). Nonetheless, the hypnotists had asked leading questions, and these false memories had come to be perceived as quite real by the subjects. As a result of hypnotic sessions, these subjects had to face the troubling consequences of having false memories of traumatic events that, in fact, had never happened to them.

The same risk is present in past life regressions. In these therapies, a traumatic false memory from a previous life may be implanted in the subject, causing significant harm. Hence, by the principle of non-malfeasance, past life regressions are not ethical. Furthermore, the time and resources wasted on past life regressions could be better allocated to therapies that are far more efficient, especially in the treatment of phobias. Cognitive behavioral therapy (CBT) is an evidence-based approach that is quite efficient in treating mood and anxiety disorders (including specific phobias). It is unethical to propose to a patient to go back to a previous life to come to terms with a traumatic event in order to treat a phobia, when in fact, it is much more efficient to do so through some of the techniques in behavioral therapy (such as flooding or systematic desensitization).
